# Reliability of lateral wall-thickness measurement in trochanteric fractures and parameters associated with lateral wall integrity

**DOI:** 10.2478/abm-2024-0005

**Published:** 2024-03-20

**Authors:** Chindarat Ratanakornphan, Thanachart Pikulhom, Somjet Jenvorapoj, Chavarin Amarase, Saran Tantavisut

**Affiliations:** Department of Radiology, King Chulalongkorn Memorial Hospital, The Thai Red Cross Society, Bangkok 10330, Thailand; Department of Orthopaedics, King Chulalongkorn Memorial Hospital, The Thai Red Cross Society, Bangkok 10330, Thailand

**Keywords:** AO/OTA classification, fracture parameter, hip fractures, lateral wall thickness, reliability

## Abstract

**Background:**

The AO Foundation/Orthopaedic Trauma Association (AO/OTA) introduced a new trochanteric fracture classification in January 2018, concerning the lateral wall integrity. It suggested the intramedullary nail fixation in patients with an incompetent lateral wall fracture.

**Objective:**

To determine the reliability of lateral wall-thickness measurement and the fracture parameters associated with lateral wall integrity.

**Methods:**

This retrospective study evaluated patients with an intertrochanteric fracture who had had surgery in King Chulalongkorn Memorial Hospital between January 2014 and January 2019. The lateral wall was measured by anteroposterior plain radiography by four raters, two times each. The demographic data and fracture parameters were assessed and compared with respect to lateral wall integrity.

**Result:**

In a total of 236 femurs and 232 patients having the 2018 AO/OTA-specified 31A1 and 31A2 intertrochanteric fractures, the lateral wall-thickness measurement showed excellent inter-rater reliability at 0.944 (0.927–0.957) and good-to-excellent intra-rater reliability ranging from 0.835 to 0.972. The parameters associated with lateral wall incompetence as per the multivariate logistic regression analysis were fracture angle (odds ratio [OR] = 0.95), distal greater trochanter involvement (OR = 9.47), and fragments at the intertrochanter area (OR = 4.49) and at the lesser trochanter (OR = 2.6).

**Conclusion:**

Some of the parameters related to trochanteric fractures are associated with lateral wall incompetence. Lateral wall-thickness measurement is a reproducible method, which has been suggested for use by the AO/OTA 2018 classification. It is easy to use and can help select the appropriate treatment for intertrochanteric fracture patients.

The AO Foundation/Orthopaedic Trauma Association (AO/OTA) Fracture and Dislocation Classification Compendium introduced a new trochanteric fracture classification in January 2018, the previous edition having been introduced in 2007. The major difference between the 2007 and 2018 classifications is the significance of the lateral wall thickness of the femur. The 2007 classification divides the fractures into the simple pertrochanteric fracture (31A1), the multifragmentary pertrochanteric fracture (31A2), regardless of the lateral wall thickness, and the intertrochanteric fracture (31A3). However, in the 2018 edition, trochanteric fractures are classified as pertrochanteric fractures with intact lateral wall (31A1), pertrochanteric fractures with incompetent lateral wall (31A2), and inter-trochanteric (reverse obliquity) fractures (31A3) [1, 2]. The lateral wall thickness is measured by a distance in millimeters drawn from a fixed point 3 cm below the innominate tubercle of the greater trochanter and angled 135° upward to the mid fracture line (**Figure 1**). The lateral wall having a thickness of <20.5 mm is determined as an incompetent lateral wall, which is associated with post-operative fractures if the patient has had dynamic hip screw fixation alone [3, 4, 5].

**Figure 1.**
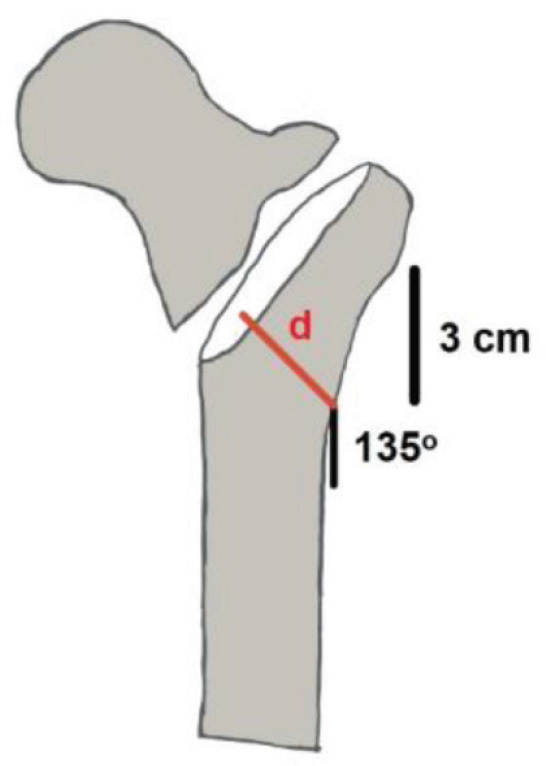
Illustration of the lateral wall-thickness measurement (d). It is defined as the distance in millimeter (mm) from a reference point that is located 3 cm below the innominate tubercle of the greater trochanter and angled at 135° upward to the midline between the two cortices of the fracture.

Our study's aims are to determine the reliability of lateral wall-thickness measurement in intertrochanteric fractures and the parameters that predict the lateral wall integrity.

## Methods

### Patient selection

There are 267 femurs and 263 patients with intertrochanteric fractures, who had had surgery in King Chulalongkorn Memorial Hospital between January 2014 and January 2019. The inclusion criteria are patients aged >18 years and the associated 2018 AO/OTA classification 31A1 and 31A2. The exclusion criteria are non-traumatic fractures (N = 1), a previous ipsilateral trochanteric region, associated fractures in the ipsi-lateral femur (N = 3), and the absence of preoperative plain radiography (N = 2). A total of 236 femurs and 232 patients were included in the study. No informed consent was obtained because the research is a retrospective descriptive study. No new imaging has been obtained in this study.

This study was approved by the Institutional Review Board of the Faculty of Medicine, Chulalongkorn University (certificate of approval no. 956/2020).

### Data collection

The medical records and the previous imaging of the patients were retrospectively reviewed. The age, gender, height, weight, BMI, BMD, side of fracture, type of fracture as per the 2018 AO/OTA classification, type of surgery, lateral wall thickness, fracture length, fracture angle, greater trochanteric involvement, and fragment site were collected.

The lateral wall thickness was measured two separate times at least 2 months apart by the radiology resident and orthopedic resident (rater 2,3—T.P., S.J.), the musculoskeletal radiologist (rater 1, C.R.), and the orthopedic surgeon (rater 4, C.A.) in an anteroposterior view of the preoperative hip or pelvis plain radiography. The raters are blinded from each other's measurements.

**Figure 2.**
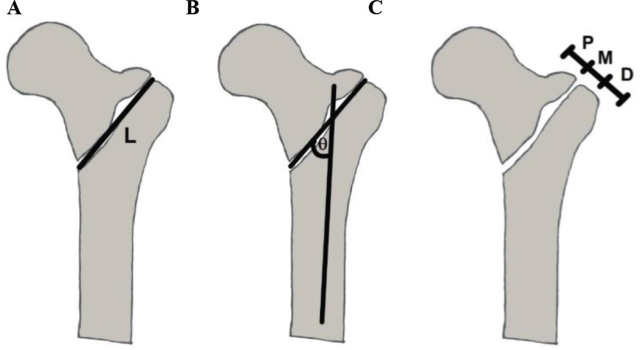
(**A**) Illustration of the fracture length (L), (**B**) fracture angle (θ), and (**C**) the greater trochanter involvement (proximal, middle, and distal).

**Figure 3.**
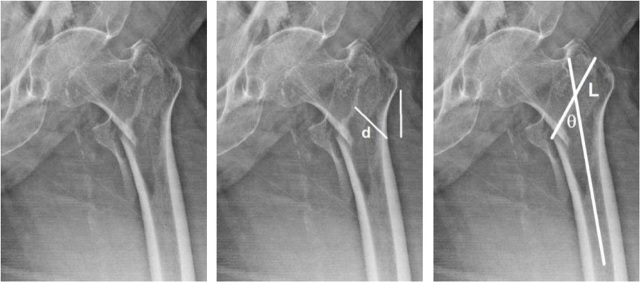
Patients with AO 31A1.3 classification intertrochanteric fracture. Lateral wall thickness = 26.34 mm, Fracture length = 6.02 cm, Fracture angle = 47°, Greater trochanter involvement at middle one–third, Fragment at lesser trochanter.

**Figure 4.**
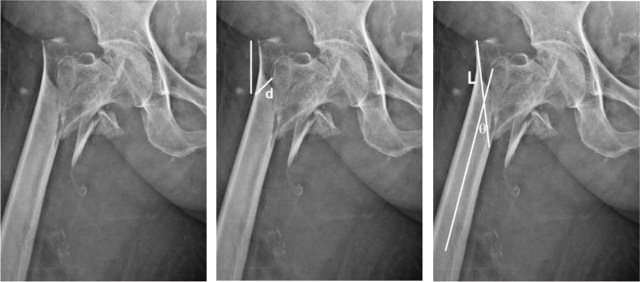
Patient with AO 31A2.3 classification intertrochanteric fracture (lateral wall incompetence). Lateral wall thickness = 14.22 mm. Fracture length = 8.01 cm. Fracture angle = 22°. Greater trochanter involvement at distal one-third, Fragment at greater and lesser trochanters.

The fracture length is measured from the length of the straight line drawn between the breaking point of the medial and lateral cortices. The fracture line angle is the angle between the femoral shaft alignment and the straight line between the breaking point of the lateral and medial cortexes. The area of involvement of the greater trochanter is divided into the proximal, middle, and distal one-third by a line from the superior to the inferior aspects of the greater trochanter. The sites of the bony fragment are the greater trochanter, the intertrochanter, and the lesser trochanter [6] as shown in **Figures 2–4**.

### Statistical analysis

The mean, SD, and independent *t*-tests were used to analyze the continuous data. Chi-squared tests was used to analyze nominal data. Univariate and multivariate binary logistic regressions were used to estimate the odds ratio (OR) to predict lateral wall integrity. The intraclass correlation coefficient (ICC) was used to analyze inter- and intra-rater reproducibility. The reliability was considered as excellent (ICC ≥0.9), good (0.9> ICC ≥0.75), fair (0.75> ICC ≥0.50), or poor (ICC <0.50) [7]. The *P*-value of <0.05 was considered as statistically significant. All data were analyzed using SPSS 22.0 (IBM).

## Results

There were 89 males (38.36%) and 143 females (61.64%) in the study having a mean age of 77.64 years (SD = 12.84). Of the 236 femurs, there were 115 right-sided fractures (48.73%) and 121 left-sided fractures (51.27%). The lateral wall status was classified according to the 2018 AO/OTA classification; 164 femurs (69.49%) were present in the intact lateral wall group (31A1.2 = 63 [26.69%], 31A1.3 = 101 [42.8%]) and 72 femurs (30.51%) were present in the incompetent lateral wall group (31A2 = 7 [2.97%], 31A2.2 = 30 [12.71%], 31A2.3 = 35 [14.83%]). The height, weight, and BMI had no significant differences between the two groups. In total, 103 cases had BMD data for the total femur and femoral neck, which were not significantly different between the two groups (*P* = 0.055, 0.388). The mean T scores of all the femurs in the intact and incompetent groups are −2.26 (SD = 1.11) and −2.70 (SD = 0.83), respectively. The mean T scores of the femoral necks in the intact group and the incompetent groups are −2.87 (SD = 1.09) and −3.06 (SD = 0.78), respectively. The age, gender, height, weight, and BMI were also insignificant between the intact and incompetent lateral wall groups as shown in **Table 1**.

**Table 1.** Demographic data for the intact and incompetent lateral wall groups

	**Intact (>20.5 mm)**	**Incompetent (≤20.5 mm)**	***P***
N	164	72	
Age (years)	76.98 ± 12.91	79.22 ± 12.542	0.221
Range (years)	24–99	25–99	
Gender			
Male	69 (42.4%)	21 (29.2%)	0.06
Female	95 (57.6%)	51 (70.8%)	
Side of fracture			
Right	82 (50%)	33 (45.8%)	
Left	82 (50%)	39 (54.2%)	
Height (cm)	159.42 ± 8.67	156.19 ± 8.35	0.461
Weight (kg)	57.00 ± 12.00	53.08 ± 10.72	0.207
BMI (kg/m^2^)	22.32 ± 3.76	21.65 ± 3.48	0.397
BMD (T score)			
Total femur	−2.26 ± 1.11	−2.70 ± 0.83	0.055
Femoral neck	−2.87 ± 1.09	−3.06 ± 0.78	0.388

The causes of fracture are: falling, 223 cases (94.49%) motorcycle accidents, 9 cases (3.81%); and bicycle accident, 4 cases (1.69%). The patients were treated with a dynamic hip screw—41 cases (17.37%), intramedullary nail—143 cases (60.59%), hip hemiarthroplasty—51 cases (21.61%), and plate and screw—2 cases (0.85%).

The mean lateral wall thickness in the intact group was 28.57 mm (SD = 4.87), which is significantly >18.11 mm (SD = 3.69) in the incompetent group (*P* < 0.0001). The mean fracture length was not statistically significant between the intact and incompetent groups, 6.51 cm (SD = 1.00) and 6.83 cm (SD = 1.59), respectively (*P* = 0.067). The fracture line angle of the intact group was significantly higher than that of the incompetent group, 40.35° (SD = 10.32) and 35.71° (SD = 12.86), respectively (*P* = 0.004). The greater trochanter involvement and fragment sites were also statistically different between the two groups (**Table 2**).

**Table 2.** Fracture parameters for the intact and incompetent lateral wall groups

**Parameters**	**Intact (>20.5 mm)**	**Incompetent (≤20.5 mm)**	***P***
Lateral wall thickness (mm)	28.57 ± 4.87	18.11 ± 3.69	<0.0001
Fracture length (cm)	6.51 ± 1.00	6.83 ± 1.59	0.067
Fracture angle (degree)	40.35 ± 10.32	35.71 ± 12.86	0.004
Greater trochanter	N	N	<0.0001
involvement	87 (53.05%)	39 (54.17%)	
Proximal	64 (39.02%)	11 (15.28%)	
Middle	13 (7.93%)	22 (30.56%)	
Distal			
Fragment	N	N	
Greater trochanter	34	28	0.004
Intertrochanter	25	31	<0.0001
Lesser trochanter	94	61	<0.0001

The inter-rater ICC was 0.944 (95% CI = 0.927–0.957), indicating excellent reliability between the four raters. The intra-rater reliability was good to excellent and was from 0.835 to 0.972 with 95% CI = 0.792–0.978 (**Table 3**).

**Table 3.** Intra-rater reliability and inter-rater reliability of lateral wall-thickness measurement

**Rater**	**Intra-rater**		**Inter-rater**	

	**ICC**	**95% CI**	**ICC**	**95% CI**
Rater 1	0.972	0.963–0.978	0.944	0.927–0.957
Rater 2	0.961	0.950–0.970		
Rater 3	0.835	0.792–0.870		
Rater 4	0.882	0.850–0.908		

ICC, Intra-class correlation coefficient.

In univariate binary logistic regression, the parameters with a statistically significant odds ratio associated with lateral wall incompetence were total femur BMD (OR = 0.33), fracture angle (OR = 0.96), greater trochanter involvement at middle (OR = 0.38) and distal (OR = 3.78), and the fragments at the intertrochanter area (OR = 3.54) and lesser trochanter (OR = 3.12) (**Table 4**).

**Table 4.** Univariate analysis of the odds ratio for the incompetent group

**Parameter**	**Odds ratio for incompetent**	***P***	**95% CI**
BMI	0.95	0.198	0.880–1.027
BMD T score			
Total femur	0.33	0.02	0.129–0.842
Femoral neck	2.27	0.09	0.877–5.864
Fracture length	1.23	0.075	0.979–1.547
Fracture angle	0.96	0.004	0.936–0.988
Greater trochanter			
involvement	0.38	0.001	0.182–0.806
Middle	3.78	<0.0001	1.726–8.258
Distal			
Fragment			
Greater trochanter	0.79	0.583	0.341–1.832
Intertrochanter	3.54	0.003	1.539–8.133
Lesser trochanter	3.12	0.003	1.461–6.667

There are 230 cases in the multivariate analysis with the exclusion of the BMD data. The statistically significant odd ratios associated with lateral wall incompetence were fracture angle (OR = 0.95), distal greater trochanter involvement (OR = 9.47), and the fragments at the intertrochanter area (OR = 4.49) and lesser trochanter (OR = 2.6) (**Table 5**).

**Table 5.** Multivariate analysis of the odds ratio for the incompetent group

**Parameter**	**Odd ratio for incompetent**	***P***	**95% CI**
Fracture angle	0.95	0.001	0.921–0.978
Greater trochanter			
involvement	9.47	<0.0001	3.517–25.475
Distal			
Fragment			
Intertrochanter	4.49	<0.0001	2.046–9.856
Lesser	2.6	0.026	1.12–6.036

## Discussion

Our study showed that the reliability of lateral wall-thickness measurements in an intertrochanteric fracture was excellent for inter-rater reliability (ICC = 0.944) and good to excellent for intra-rater reliability (ICC = 0.835–0.972). Therefore, the lateral wall-thickness measurement is reproducible per the experience of the raters (in our study, they included an orthopedic surgeon, musculoskeletal radiologist, and residents of the orthopedic and radiology departments). This can be useful in classifying the intertrochanteric fracture group according to the 2018 AO/OTA classification. Chan et al. [8] found that the 2018 AO/OTA intertrochanteric fracture classification had moderate inter-rater reliability in group classification (Cohen's kappa = 0.479) and fair inter-rater reliability in subgroup classification (Cohen's kappa = 0.376). The raters consisted of two orthopedic surgeons and four residents. However, they did not demonstrate the reliability of the lateral wall-thickness measurement. Further studies may be needed to establish the reliability of the 2018 AO/OTA intertrochanteric fracture classification and lateral wall-thickness measurement.

Our study further describes the fracture parameters that are associated with lateral wall incompetence. We found that fracture angle (OR = 0.95), distal greater trochanter involvement of the fracture (OR = 9.47), and fragments at the intertro-chanter area (OR = 4.49) and the lesser trochanter (OR = 2.6) are associated with lateral wall incompetence in intertrochanteric fracture. These findings will help when classifying patients. If the fracture has distal greater trochanter involvement or the fragments at the intertrochanter area or the lesser tro-chanter, it is more likely to be lateral wall incompetence that suggests unstable type of fracture and suitable for intramedullary nail fixation. On the contrary, if the fracture has a high fracture angle, the lateral wall has more chance to be intact. Stable intertrochanteric fracture is currently treated with either dynamic hip screw or intramedullary nail fixation.

Due to the retrospective nature of the study, some plain radiographs had a poor position or inadequate traction, obscuring the fracture line that could cause errors in the measurements and thus, affect the reliability. We reduced the errors by selecting the best possible image, and all the raters measured the same film. We also excluded the BMD data in the multivariate analysis because there are about 66% of the cases with missing data. This could confound the multivariate analysis.

## Conclusion

In summary, lateral wall-thickness measurements of the AO/OTA 2018 classified intertrochanteric fractures show excellent inter-rater reliability. Fracture angle, distal greater trochanter involvement, and the fragments at the intertrochanteric region and the lesser trochanter are associated with lateral wall incompetence in intertrochanteric fractures.
